# Salvage radiotherapy in patients with local recurrent esophageal cancer after radical radiochemotherapy

**DOI:** 10.1186/s13014-015-0358-z

**Published:** 2015-02-27

**Authors:** Zhi-guo Zhou, Chan-jun Zhen, Wen-wen Bai, Ping Zhang, Xue-ying Qiao, Jun-li Liang, Xian-shu Gao, Shuo-shuo Wang

**Affiliations:** Department of Radiation Oncology, Peking University First Hospital, Beijing, 100034 China; Department of Radiation Oncology, Fourth Hospital of Hebei Medical University, Shijiazhuang, Hebei 050011 China

**Keywords:** Salvage radiotherapy, Esophagus, Squamous cell carcinoma, Local recurrence

## Abstract

**Purpose:**

The aim of this study was to evaluate the salvage radiotherapy outcome in patients with local recurrent esophageal cancer after radical radiochemotherapy (RCT).

**Methods:**

A total of 114 patients with local recurrent esophageal squamous cell carcinoma after initial radical RCT were retrospectively analyzed. Fifty-five (55) patients belonged to the salvage radiotherapy group (SR group) and 59 patients to the non-salvage radiotherapy group (NSR group).

**Results:**

The median survival time after-recurrence was 4 months in all patients. The 1, 2, 3 year overall survival (OS) rates were 83.6%, 41.8% and 21.8% respectively in the SR group, and 57.6%, 16.9%, and 8.5% in the NSR group. The 6-month and 1-year survival rates after-recurrence were 41.8% and 16.4% respectively in the SR group, and 11.9% and 3.4% respectively in the NSR group. A salvage radiation dose > 50 Gy after initial radical RCT, improved the survival of patients with local recurrent esophageal cancer. Three patients (5.45%) from the SR group showed more than 3-grade radiation pneumonitis. In addition, esophageal fistula/perforation was observed in 11 cases (20.0%) in the SR group and in 8 cases (13.6%) in the NSR group.

**Conclusions:**

Salvage treatment after definitive RCT may improve the overall survival and survival after-recurrence of patients with local recurrent esophageal cancer.

## Introduction

Loco-regional recurrence is still the major type of treatment failure in patients with esophageal cancer after definitive radiotherapy (RT) or radiochemotherapy (RCT). The recurrence rate after radical radiotherapy, radiochemotherapy and surgery is more than 70% [1-2]. Once recurrence occurs, the 5-year survival rate became worse [3-4]. Treatments such as salvage surgery, radiotherapy/radiochemotherapy and chemotherapy are usually carried out on patients with recurrent esophageal cancer. However, these treatments on recurrent esophageal cancer (REC) report similar unsatisfactory results with regard to survival [4-6]. Moreover, the patients in those studies accepted surgery as an initial treatment [4-5], while rarely they accepted chemoradiotherapy. Although the effectiveness of radiotherapy as a primary treatment of esophageal cancer has been adequately demonstrated [7-9], and re-irradiation has also been proven to be feasible and effective in other tumors [10-11], the advantage of re-radiotherapy as a salvage treatment of REC after RCT is uncertain. In addition, no further studies were performed to address the effectiveness and feasibility of salvage radiotherapy for those patients with local recurrent esophageal cancer after primary radical RT/RCT [12].

The local recurrence in the primary tumor bed after radiotherapy is more often found in esophageal cancer and represents the most important example of radiotherapy failure [13-15]. The role of salvage treatments or palliative therapies (such as chemotherapy, esophagectomy, stentplacement, feedingtube placement) in local recurrent esophageal cancer after radiation remains controversial [16]. Thus, the purpose of this study was to retrospectively evaluate the salvage radiotherapy outcome after radiochemotherapy in patients with local recurrent esophageal cancer.

## Materials and methods

### General clinical data

Hundred fourteen (114) patients with local recurrent esophageal cancer after radical RCT were retrospectively analyzed from December 2003 to January 2012. The following criteria were used to recruit the patients: 1) patients with esophageal cancer who had received definitive RCT treatment; 2) patients with pathologically confirmed squamous cell carcinoma; 3) patients with local recurrence confirmed by pathological analysis, without simultaneous local regional lymph nodes recurrence or distant recurrence; 4) patients with no salvage esophagectomy treatment after recurrence; 5) patients without any other serious medical illness except esophageal cancer. Local recurrence in the primary tumor bed was diagnosed by computed tomography (CT) and upper gastrointestinal endoscopy. All the patients were divided into two groups according to the administration of the salvage radiotherapy: 55 patients in the salvage radiotherapy group (SR group) and the remaining patients in the non-salvage radiotherapy group (NSR group). Patients belonging to NSR group were subjected to chemotherapy, gastrostomy, stent implantation and feeding tube support care. Patients’ basic and clinical profiles are summarized in Table 1.

**Table 1:** **Patients characteristics (N = 114)**

**Characteristics**	**SR group N (%)**	**NSR group N (%)**	**P value**
Gender			
Female	28 (50.9)	31 (52.5)	0.862
Male	27 (49.1)	28 (47.5)	
Age (years) Mean ± SD	66.8 ± 8.8	63.7 ± 8.6	0.062
Location of tumor			
Upper	32 (58.2)	41 (69.5)	0.192
Middle	22 (40.0)	15 (25.4)	
Lower	1 (1.8)	3 (5.1)	
Initial length (cm) Mean ± SD	4.9 ± 2.0	5.7 ± 2.5	0.046
Initial ECOG-PS			
0-I	46 (83.6)	52 (88.1)	0.523
II	8 (14.6)	7 (11.9)	
III	1 (1.8)	0 (0)	
Initial clinical stage^#^			
I-II	40 (72.7)	36 (61.0)	0.185
III	15 (27.3)	23 (39.0)	
Initial radiation dose (Gy) Mean ± SD	61.2 ± 5.4	62.1.6 ± 2.6	0.262
TRS time			
≤12 months	24 (43.6)	38 (64.4)	0.026
>12 months	31 (56.4)	21 (35.6)	
Salvage radiation dose			
≤50 Gy	24 (43.6)	NA*	NA*
>50 Gy	31 (56.4)		
Mean ± SD (Gy)	51.9 ± 10.3		

^#^According to TNM classification 6th edition,*NA: not-available.

### Treatment

All patients were initially treated with high-energy photons using 6 MV linear accelerators, 1.8–2.0 Gy per fraction, 5 days/week. The initial radical radiation dose was defined as more than 54 Gy. Initial radiation therapy was performed using conventional two-dimensional or conformal three-dimensional planning, followed by a cisplatin-based chemotherapy regimen.

After the initial treatment described above, the SR group patients received a three-dimensional conformal radiotherapy (3D-CRT) or an intensity-modulated radiotherapy (IMRT). The gross tumor volume (GTV) after-recurrence was defined as the region of recurrence determined by endoscopic investigation and CT scan. The clinical target volume (CTV) was defined as the GTV plus a margin of 0.8–1.0 cm on each side and 1.5-2.0 cm above and below the tumor mass. The planning target volume (PTV) was defined as the CTV plus a 0.5 cm margin in all directions. Salvage radiotherapy was carried out with a median dose of 54 Gy (range 18–66 Gy), 1.8–2.0 Gy per fraction, 5 days/week. The initial irradiation dose limit for the spinal cord was represented by a maximum dose of less than 45 Gy, and for salvage radiotherapy no more than 20 Gy. Regarding the lungs, the mean dose and V20 were limited within 20 Gy and 30% respectively in the first treatment, while the V20 was less than 25% after-recurrence treatment. Toxicities were evaluated according to the National Cancer Institute Common Toxicity Criteria version 3.0.

### Statistical analysis

Statistical analyses were performed using the SPSS software (version 18.0). The two treatment groups were compared to base-line characteristics, with the *t*-test and the *X*^2^ test used for continuous and categorical variables, respectively. The overall survival (OS) time was considered from the start of the treatment to the date of death or last follow-up. The time of recurrence survival (TRS) was measured from the first day of the initial treatment to the day the recurrence was pathologically confirmed. The after-recurrence survival (ARS) time was calculated from the date of relapse to the date of death or last follow-up. The rates of survival curves, depending on different factors, were calculated using the Kaplan-Meier analysis method, and were compared using a log-rank test. A p value < 0.05 was considered statistically significant. Cox’s proportional hazards regression model was used to determine the effect of multiple factors on survival.

## Results

The last follow-up was in June 2013. The median follow-up period was 20 months (range 8–70 months) in all patients. Only the recurrence of the tumor in the primary tumor bed was included in this study, and simultaneous local regional lymph nodes recurrence was not taken into account. Forty seven patients (47) of the 55 belonging to the SR group completed the salvage radiotherapy plan.

All patients died during the follow-up period. The median overall survival time of the whole cohort was 17 months (range 7–65 months). The 1, 2, 3 year overall survival rates in all patients were 70.2%, 28.9% and 14.9%, respectively. The median time of recurrence survival (TRS) after initial treatment was 12 months (range: 6–56 months) in the whole cohort. However the median survival time after-recurrence (ARS) was as short as 4 months. The 1, 2, and 3 year local control rates of all the patients were 45.6%, 14.9% and 5.3% respectively.

**Figure 1:**
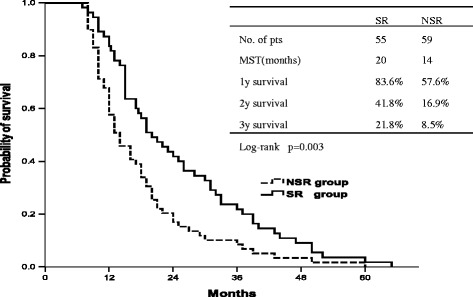
**Overall survival (OS) curve of NSR group and SR group.**

**Figure 2:**
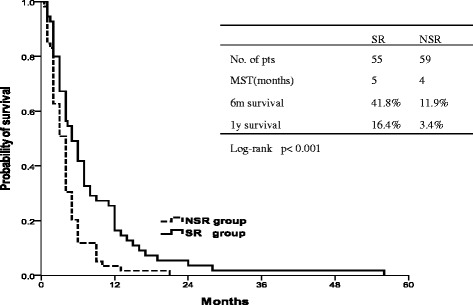
**After-recurrence survival (ARS) in the SR and in the NSR group.** The survival is significantly increased in the SR group compared to the NSR group (p < 0.001).

The 1, 2, 3 year overall survival rates were 83.6%, 41.8% and 21.8%, respectively in the SR group, and the median survival time was 20 months. The 1, 2, 3 year overall survival rates were 57.6%, 16.9%, and 8.5%, respectively in the NSR group (Figure 1), and the median survival time was 14 months. SR group patients showed better outcomes with a significant improvement of the OS (p = 0.003), compared to the NSR group. The 6-months and 1-year after-recurrence survival (ARS) rates were 41.8% and 16.4% respectively in the SR group, and 11.9% and 3.4%, respectively in the NSR group. The survival is significantly increased in the SR group respect the NSR group (p < 0.001) (Figure 2). The TRS in the SR group (median 14.0 months, 95% CI 11.3-16.7 months) was longer than the one in the NSR group (median 10.0 months, 95% CI 11.3-16.7 months), but no significant difference was found between the two groups (p = 0.062).

**Figure 3:**
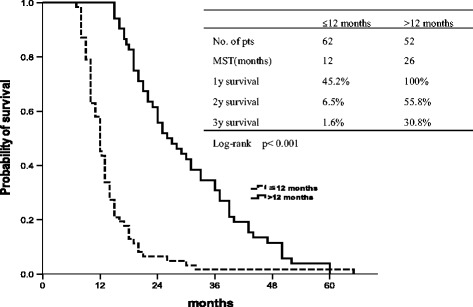
**Overall survival with late recurrence (>12 months) and early recurrence (≤12 months).**

The median overall survival rate of patients with late recurrence (>12 months) was 26 months, and the median overall survival time of patients with early recurrence was 12 months (p < 0.001) (Figure 3). Patients with late recurrence (>12 months) showed a median after-recurrence survival (ARS) of 5 months instead of 3 months for those experiencing early relapse (≤12 months), with a statistical value close to 0.05 although not significant (p = 0.061).

**Figure 4:**
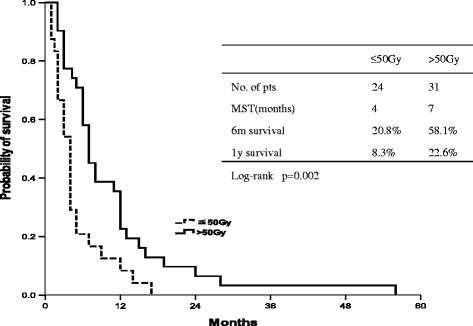
**After-recurrence survival (ARS) using radiation doses ≤50 Gy and >50 Gy.**

In sub-group analysis, the salvage irradiation dose had a clear impact on the outcomes in the SR group patients. Patients receiving more than 50 Gy irradiation dose (median 7.0 months, 95% CI 5.2-8.8 months) showed significantly prolonged ARS than those who received an irradiation dose of 50 Gy or less (median 4.0 months, 95% CI 3.0-4.9 months) (p = 0.02) (Figure 4).

Multivariate factor analysis for overall survival revealed that salvage radiotherapy, location of tumor and TRS time may be considered significant predictors (Table 1). Significant and favorable factors for ARS were represented only by salvage radiotherapy (P < 0.003).

By the end of the follow-up, radiation myelitis was not observed in the SR group. Three patients (5.45%) with radiation pneumonitis of more than grade 3 were evaluated in the SR group, and two of them died of severe lung infection. Nineteen (19) patients manifested esophageal fistula/perforation, which appeared after local recurrence. All of them manifested a toxicity grade of more than 4. The esophageal fistula/perforation was observed in 11 cases (20.0%) and in 8 cases (13.6%), in the SR and in the NSR group, respectively, and there was no significant difference between the two groups (p = 0.357). The profiles of the patients with fistula/perforation in the SR group are summarized in Table 2. The causes of death for all patients are shown in Table 3. The causes of death for all patients are shown in Table 4. No significant difference was found between the two groups (p = 0.801).

**Table 2:** **Prognostic factors by log-rank test and univariate survival analysis**

**Characteristics**	**Number**	**Median OS(months)**	**Log-rank test p value**	**Univariate analysis p value**
Gender				
Female	59	18	0.975	0.573
Male	55	16		
Age (years)				
<60	33	15	0.536	0.310
≥60	81	18		
Location				
Upper	73	19	0.001	0.02
Middle	37	15		
Lower	4	12.3		
Initial length (cm)				
≤5 cm	72	17	0.806	0.381
>5 cm	42	17.5		
Initial ECOG-PS				
0-I	98	17.5	0.150	0.647
II	15	13		
III	1	12		
Initial clinical stage				
I-II	76	21	0.0002	0.07
III	38	10		
TRS time				
≤12 months	62	12	0.0001	0.0002
>12 months	52	26		
Group				
SR	55	20	0.003	0.001
NSR	59	14

**Table 3:** **Patients’s characteristics with fistula/perforation in the SR group (N = 55)**

**Characteristics**	**Fistula/perforation N (%)**	**No fistula/perforation N (%)**	**P value**
Total radiation dose (Gy)	112.9 ± 14.9	113.0 ± 11.8	0.918
Initial radiation dose (Gy)	62.9 ± 3.8	60.7 ± 5.5	0.686
Salvage radiation dose (Gy)	50.0 ± 12.2	52.4 ± 9.9	0.746
≤50Gy	5 (45.5%)	19 (43.2%)	0.892
>50Gy	6 (54.5%)	25 (56.8%)	
Time after salvage radiotherapy end			
≤3 months	6 (54.5%)	NA*	NA*
>3 months	5 (45.5%)		

*NA: not-available.

**Table 4:** **List of death causes (N = 114)**

**Cause of death**	**SR group N (%)**	**NSR group N (%)**	**P value**
Local failure	38(69.0%)	44(74.5%)	0.824
Fistula/perforation	9(16.4%)	8(13.6%)	
Local failure and metastasis	5(9.1%)	3(5.1%)	
Infection	3(5.5%)	4(6.8%)	

## Discussion

The recurrences after initial treatment in patients with esophageal cancer remain a serious challenge for clinical oncologists. In addition; the recurrences of esophageal cancer represent a very common event. The reported local recurrence after surgery is indeed 12.1%, and the lymph node metastases rate is 18.2% [11-12]. However, after radiotherapy/chemoradiotherapy, the local recurrence was as high as 78.4% and lymph node metastases were 33.3% in recurrent patients [13-15]. Some controversy exists regarding the best salvage treatment [17-20]. Salvage treatment of recurrent esophageal cancer depends on the position of the recurrence and on the initial treatment. Usually, radiation is not considered if it has already been administrated as part of the initial treatment [16-20]. Only several small size studies reported the outcome of salvage radiotherapy of local-region recurrence for patients experiencing initial radical RCT [12]. Therefore our results represent an important supplement to salvage treatment with recurrent esophageal cancer, especially regarding local primary site recurrence.

Recurrence leads to a remarkable decrease of the survival time, which is also observed in our study. The median overall survival time in our study was 17 months in the whole cohort. However, the median survival time was remarkably decreased to 4 months after recurrence. It is noticeable that more than 80% of the relapse in our study occurred in two years after irradiation, and the 2-years local control rate of all patients was 14.9%. This result was similar to that reported by Ishihara et al. [21], which showed 82% recurrence of esophageal cancer developed within 21 months of RCT.

Our data on salvage radiotherapy in recurrent patients disclosed an excited survival status. The 3-years overall survival rate was 21.8% in salvage radiotherapy patients, and the median survival time was 20 months. These are certainly excellent results compared to those reported in other studies [22,23]. Yamashita et al. [22] reported the results of radiotherapy with or without chemotherapy in patients with loco-regional recurrence of esophageal cancer after curative surgery, with median survival time of 13.8 months, and 1-year survival rate of 56%. Baxi et al. [23] reported a 2-years survival rate of 21% for all patients and median survival time of 16 months for patients with recurrence after surgery. The reason for these differences between our and other studies may be due to different baselines of initial treatment and a different position of the recurrent tumor.

Patients experiencing recurrence at 12 months or less (early recurrence) after radical RT/RCT showed a lower survival rate compared with patients experiencing a tumor recurrence after more than 12 months (late recurrence). TRS time was significant in both univariate (p = 0.0001) and multivariate analyses of survival (p = 0.0002). Our findings are in agreement with those reported by Shimada et al. and Yu et al. [24-25]. Recurrence time may be associated with the growth rate of recurrent tumors. For this reason, early recurrences might be due to fast-growing tumor cells that do not respond to the treatment [25].

Minsky et al. [9] used 50.4 Gy as a standard irradiation dose for esophageal cancer in trial INT 0123, and the higher radiation dose did not increase the survival or improved the local/regional control. However, the irradiation dose of nearly 60 Gy used for patients with recurrence after surgery, showed encouraging median OS (16–39 months)as reported in some studies [2,20,23,26]. Our study also highlighted similar results. The survival time of the patients receiving salvage RT with a dose > 50 Gy was longer than that obtained with a dose ≤50 Gy. This might suggest that a higher RT dose is able to inhibit the growth rate of recurrent esophageal cancer to some extent. Thus, a re-irradiation dose higher than 50 Gy may improve the after-recurrence survival or overall survival of these patients.

Salvage radiotherapy was completed in 85.5% (47/55) of the SR group patients, and most patients were tolerant to re-irradiation. No patients with radiation myelitis were observed in the SR group. However, we did not have any evidence of spinal cord damage, since most of the patients died within one year after recurrence. The observation time was probably not long enough. Three cases showed radiation pneumonitis of more than 3-grade, and all of them received a RT dose more than 50 Gy. Moreover, 11 patients with the esophageal fistula/perforation were observed in the SR group. It is unclear whether these adverse effects were associated with salvage radiation or not. Therefore, the suitable irradiation dose for recurrent esophageal cancer remains uncertain and requires further investigations as some researchers suggested [20].

Although this work is a retrospective analysis with a limited cohort of patients, we elucidated the survival benefit of radiotherapy for patients with recurrent disease after RCT, which was barely reported in previous studies. In addition, our results suggest that salvage radiotherapy may improve survival in those patients, and may have the potential to enhance radiation oncologists’ treatment strategy.

## Conclusion

Salvage radiation therapy appear to be a promising treatment in the management of local tumor bed recurrence of esophageal cancer after definitive radiochemotherapy. The use of this salvage treatment may improve the overall survival and after-recurrence survival of these patients. A salvage radiation dose >50 Gy and late recurrence (>12 months) were associated with a better prognosis for those patients. However, attention should be paid to the esophageal fistula/perforation after salvage radiation.
